# Breast Cancer Classification with Various Optimized Deep Learning Methods

**DOI:** 10.3390/diagnostics15141751

**Published:** 2025-07-10

**Authors:** Mustafa Güler, Gamze Sart, Ömer Algorabi, Ayse Nur Adıguzel Tuylu, Yusuf Sait Türkan

**Affiliations:** 1 Engineering Sciences Department, Engineering Faculty, Istanbul University-Cerrahpasa, Istanbul 34320, Türkiye; 2 Department of Educational Sciences, Hasan Ali Yucel Faculty of Education, Istanbul University-Cerrahpasa, Istanbul 34500, Türkiye; gamze.sart@iuc.edu.tr; 3 Industrial Engineering Department, Engineering Faculty, Istanbul University-Cerrahpasa, Istanbul 34320, Türkiye; omer.algorabi@iuc.edu.tr (Ö.A.); ayse.adiguzeltuylu@iuc.edu.tr (A.N.A.T.); ysturkan@iuc.edu.tr (Y.S.T.)

**Keywords:** deep learning, breast cancer, image processing, tumor

## Abstract

**Background/Objectives:** In recent years, there has been a significant increase in the number of women with breast cancer. Breast cancer prediction is defined as a medical data analysis and image processing problem. Experts may need artificial intelligence technologies to distinguish between benign and malignant tumors in order to make decisions. When the studies in the literature are examined, it can be seen that applications of deep learning algorithms in the field of medicine have achieved very successful results. **Methods:** In this study, 11 different deep learning algorithms (Vanilla, ResNet50, ResNet152, VGG16, DenseNet152, MobileNetv2, EfficientB1, NasNet, DenseNet201, ensemble, and Tuned Model) were used. Images of pathological specimens from breast biopsies consisting of two classes, benign and malignant, were used for classification analysis. To limit the computational time and speed up the analysis process, 10,000 images, 6172 IDC-negative and 3828 IDC-positive, were selected. Of the images, 80% were used for training, 10% were used for validation, and 10% were used for testing the trained model. **Results:** The results demonstrate that DenseNet201 achieved the highest classification accuracy of 89.4%, with a precision of 88.2%, a recall of 84.1%, an F1 score of 86.1%, and an AUC score of 95.8%. **Conclusions:** In conclusion, this study highlights the potential of deep learning algorithms in breast cancer classification. Future research should focus on integrating multi-modal imaging data, refining ensemble learning methodologies, and expanding dataset diversity to further improve the classification accuracy and real-world clinical applicability.

## 1. Introduction

The breast is an important structure in the female body, both biologically and socially. Biologically, breast tissue produces milk during lactation, providing nutrition and immune support to the newborn. Breast milk is critical in the development and health of babies, as it is rich in antibodies and nutrients. In addition, as part of the hormonal system, the breast is sensitive to hormones such as estrogen and progesterone and is associated with reproductive health. In addition, the breast plays an important role in women’s body image, identity formation, and feelings of sexual attractiveness. But beyond these functions, breast health also requires great medical attention due to the importance of the early detection and treatment of diseases such as breast cancer. Breast cancer is the most frequently diagnosed cancer among women worldwide, with approximately 2.3 million new cases and 685,000 deaths reported in 2020 [1]. In the United States, about 1 in 8 women (13%) will develop invasive breast cancer during their lifetime, with an estimated 310,720 new cases expected in 2024. Although it mostly affects women, it is also seen in men. It arises from the uncontrolled growth of cells in the breast, often originating in the milk ducts or lobules. According to American Cancer Society, risk factors include genetic predispositions, such as mutations in the BRCA1 and BRCA2 genes, hormonal influences, lifestyle factors like alcohol consumption, and prolonged estrogen exposure. Early detection through regular screening, including mammography, significantly improves survival rates, while treatment options include surgery, chemotherapy, radiation, and targeted therapies depending on the cancer stage and type.

Artificial intelligence (AI) has revolutionized medical image classification, playing a critical role in diagnosing and managing various diseases, including cancer, cardiovascular conditions, and neurological disorders. By leveraging advanced algorithms, such as deep learning methods, AI systems can analyze complex imaging data with remarkable accuracy, often exceeding human performance in detecting abnormalities [2]. This capability enhances early diagnosis, enabling timely intervention and improving patient outcomes. Furthermore, AI streamlines workflows by reducing the burden on radiologists, increasing efficiency, and standardizing interpretations. In areas with limited healthcare access, AI-powered tools can democratize diagnostic services, offering lifesaving insights where skilled professionals are scarce.

Deep learning is an approach to machine learning based on neural networks. Deep learning networks consist of multiple layers, and in this complex architecture, each neuron in the layer is connected to the neuron in the next layer with weighted connections [3]. Recently, deep learning has been used in many areas, especially for complex, challenging problems such as image recognition [4], emotional intelligence [5], speech recognition [6], self-driving cars [7], and Industry 4.0 [8].

A convolutional neural network (CNN) is a deep learning method that can automatically and adaptively learn spatial hierarchies of features from input images, ranging from simple edges and textures to complex objects. CNNs employ convolutional layers to preserve spatial relationships between pixels, pooling layers to reduce dimensionality, and fully connected layers to classify features [9]. This architectural design reduces the necessity for manual feature extraction, thereby rendering CNNs particularly effective for complex datasets.

CNNs have become essential in medical image recognition due to their exceptional ability to analyze and interpret visual data. Unlike traditional machine learning models, CNNs automatically extract hierarchical features from images, making them highly effective for tasks like detecting tumors, segmenting tissues, and classifying medical conditions in X-rays, MRIs, CT scans, and histopathological images. Their architecture, designed to process spatial data, leverages convolutional layers to identify intricate patterns and features such as edges, textures, and anomalies. This capability reduces the reliance on manual feature engineering and enhances accuracy and speed. For instance, CNNs are widely used in detecting cancer [10] or diagnosing retinal diseases [11] from images, often achieving performance comparable to or better than that of expert radiologists. Their adaptability and scalability also allow for integration into large datasets, continuously improving their diagnostic accuracy and supporting early intervention, which is crucial in saving lives.

This study addresses the issue of breast cancer classification using convolutional neural network (CNN) models. Following the introduction, a review of the literature on the applications of artificial intelligence in healthcare in recent years is presented. The manuscript provides an overview of the dataset, the study’s flow chart, and the CNN models used and their architectures. It also presents findings and comparative analyses in both tabular and graphical formats, exploring the study’s discoveries and limitations. Finally, it concludes with a summary of the study’s significance, along with suggestions for future research and potential applications.

## 2. Related Studies

When studies in which artificial intelligence technology is applied, especially in the field of medicine, are examined, it can be seen that a significant amount of successful research has been conducted. As a result of the development of image processing studies, recently, the interpretation of findings obtained from radiological images has become quite easy. This knowledge is very important for both specialists and patients to be able to make quick decisions and take action.

When we take a look at similar studies, we can see that there are studies in the literature that develop deep learning-based models for breast cancer diagnosis. A convolutional neural network (CNN) algorithm and dimensionality reduction with PCA (Principal Component Analysis) were used in one study [12]. Direct predictions from images were made using the end-to-end GoogleNet model, and more advanced predictions were produced by feature extraction from images with the GoogleNet-LDA model [13]. The advantages and limitations of various medical imaging modalities, including digital mammography (DMG), ultrasound, magnetic resonance imaging (MRI), and biopsy, were examined [14]. Logistic regression, random forest, support vector machines (SVMs), AdaBoost, bagging (bootstrap aggregation), voting classifiers, and a deep learning-based Xception model were employed to classify tumors, and the outcomes were compared [15]. A novel method called the “Deep Learning Assisted Efficient AdaBoost Algorithm” (DLA-EABA), which combines CNNs with the AdaBoost algorithm, was introduced [16]. Deep learning techniques were applied for tumor detection, localization, and characterization using MR data [17]. A Bayesian YOLOv4 network was developed for tumor detection in automated breast ultrasound (ABUS) images [18]. Two SegNet architectures with “skip connections” were combined in the Connected-SegNets model to improve the accuracy of tumor segmentation in X-ray images and reduce false positive/negative rates [19]. BTEC-Net, MSSE-DenseNet121, and MSSE-ResNet101 models were combined to achieve high accuracy and F1 scores in classifying ultrasound images [20]. The AlexNet model was used for feature extraction, followed by the selection of important features with the Relief algorithm, and machine learning techniques such as least-squares support vector machine, KNN, random forest, and Naive Bayes were applied for disease detection and classification [21]. The BUSNet model was developed for tumor detection in breast ultrasound (US) images and was compared with other two-stage approaches such as Faster R-CNN and Cascade R-CNN [22]. The integration of ultrasound (US) technologies into deep learning (DL) models in the diagnosis and management of breast cancer was examined, and the performance of different DL models at various stages was analyzed [23]. Feature extraction was performed using GLCM and GLRLM techniques, and good classification results were achieved with support vector machines, random forests, and artificial neural networks [24]. A new deep learning approach called Efficient U-Net, which combines EfficientNet with atrous convolution (AC) blocks to address issues in the U-Net design, was introduced [25]. An Inception ResNetV2 transfer learning model was utilized in another study [26]. Random forest, Decision Tree, KNN, Logistic Regression, SVC, and Linear SVC algorithms were utilized to analyze mammography images and classify various types of cancer [27]. In their study, ResNet-50, GoogLeNet, Inception-v3, and MobileNet-v2 models were employed for classification, and the Gray Wolf Optimization (GWO) algorithm was applied for feature selection [28]. Meanwhile, the DenseNet201 model was used as a feature extractor for classifying histopathological images and subsequently combined with the XGBoost classifier [29]. Additionally, the deep breast CancerNet model was developed [30]. To address the limitations of existing models, a model inspired by GoogLeNet and residual block structures was proposed [31]. A model based on the SE-ResNet-50 architecture was built and compared with other architectures such as ResNet50, GoogleNet50 and EfficientNet B2 [32]. The “Squeeze and Excitation” method was also used to model the dependencies between feature channels, “Generalized Average Pooling” was applied instead of average pooling, and the Adam algorithm was preferred for optimization. A two-stage computer-aided diagnosis system was developed based on the classification of digital breast tomosynthesis (DBT) images using the VGG19 model with additional layers (batch normalization and pooling layers) and tumor detection using the YOLOv5-CBAM model (the YOLOv5 model combined with a Convolutional Block Attention Module (CBAM)) [33]. The MIRAI prediction model was used to predict breast cancer risk for 1–5-year periods [34]. AlexNet, ResNet18, and MobileNetV2 transfer learning models were combined to provide both speed and accuracy. They improved the image quality with Laplacian of Gaussian (LoG) and high-boost filtering. The system was also optimized by using techniques such as residual learning, deep separable convolutions, and inverse residual structure to make the system faster [35].

Machine learning and deep learning techniques have been successfully utilized in the diagnosis of various types of cancer. The use of an optimized YOLOv7 model for detecting different types of brain tumors, including meningiomas, gliomas, and pituitary tumors, in MRI images has been described [36]. This model incorporates innovative components such as the Convolutional Block Attention Module (CBAM), Spatial Pyramid Pooling Fast (SPPF+), and the Bi-directional Feature Pyramid Network (BiFPN) to enhance its performance. The combination of different imaging modalities, specifically magnetic resonance imaging (MRI) and computed tomography (CT), along with discrete cosine transform (DCT), for brain tumor diagnosis has also been explored [37]. By employing convolutional neural networks (CNNs), important features were extracted from the images, and classification was performed using methods such as support vector machines (SVMs), K-Nearest Neighbors (KNNs), and Decision Trees (DTs) [37]. A new method for the automatic analysis of MRI images using GLCM-based feature analysis and optimized CNN (Convolutional Neural Network) and LSTM (Long Short-Term Memory) algorithms for the accurate diagnosis of brain tumors has been presented [38]. A hybrid model combining the strengths of two famous convolutional neural networks (CNNs), VGG16 and ResNet50, for skin cancer diagnosis has been built [39]. Vision Transformer (ViT) and Swin Transformer models have been used on histopathological images for colon cancer diagnosis, and a new modified Swin Transformer model has been proposed [40]. Cancer Cell Detection using a Hybrid Neural Network (CCDC-HNN), an innovative hybrid deep learning method for the early detection and diagnosis of breast cancer, has also been proposed [41]. Advanced CNN-based architectures often outperform Transformers on limited datasets because the latter suffer from spatial bias (inductive bias). However, CNNs, especially optimized variants (e.g., EfficientNet, ResNet), consume fewer computational resources and have a shorter inference time compared to Transformer architectures. In addition, there is a large and mature transfer learning ecosystem for CNNs, resulting in less training time and faster prototyping. In conclusion, while Vision Transformer architectures provide flexibility and scalability in the long term, advanced CNNs still have the advantage of requiring less data and showing a lower resource consumption and more stable operation.

## 3. Materials and Methods

### 3.1. Datasets

**Figure 1:**
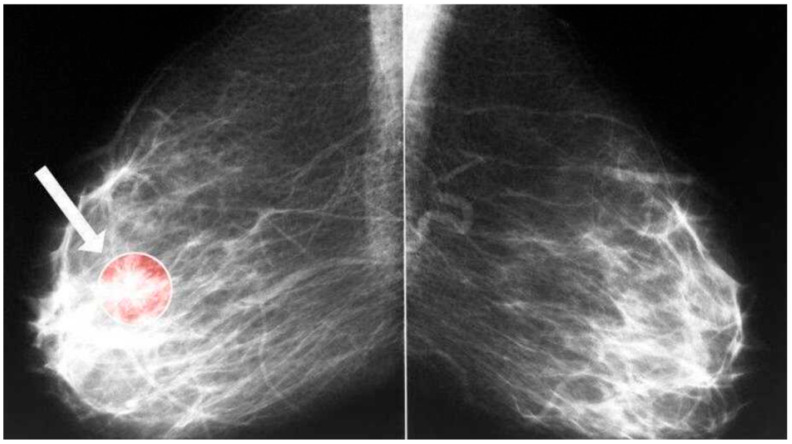
Original breast tumor image.

**Figure 2:**
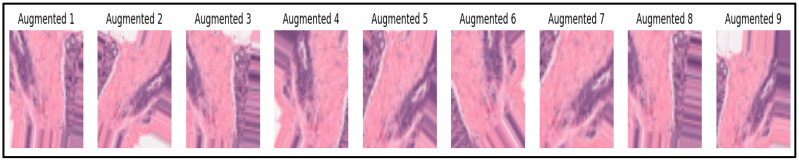
Original augmented images.

In this study, we used the open access ‘Predict IDC in Breast Cancer Histology Images’ image set created by Paul Mooney and obtained from the Kaggle platform [42]. In this image classification analysis, we used images of pathological samples from breast biopsies to classify them into 2 classes: benign or malignant. These classes are labeled as ‘Benign’ and ‘Malignant’. The original dataset contains full montage slide images of breast cancer (intraductal carcinoma (IDC)) specimens scanned with 40× zoom. Images were cropped at patches of size 50 × 50, and 277,254 patches were created; 198,738 were IDC-negative, and 78,786 were IDC-positive. To limit the computational time and speed up the analysis process, 10,000 images, 6172 IDC-negative and 3828 IDC-positive, were selected. Of the images, 80% were used for training, 10% were used for validation, and 10% were used for testing the trained model. To improve the generalization and model robustness and reduce overfitting, data augmentation was carried out before training. Each 50 × 50 patch was given to the CNN as a separate sample. The CNN predicted the class to which this patch belonged (e.g., tumor present/absent, surface smooth/distorted). In this study, pre-trained CNN models (e.g., ResNet, VGG) were used to extract features from 50 × 50 patch images, and then a decision could be made with classifiers (SVM, random forest). Also, images were normalized, and data augmentation with rotation (40°), width and height shifting (0.2), shearing (0.2), zooming (0.2), and horizontal flipping was carried out. An example of an original image and 9 augmented images can be seen in Figure 1 and Figure 2. Figure 1 also shows an image of the breast with and without a tumor.

**Figure 3:**
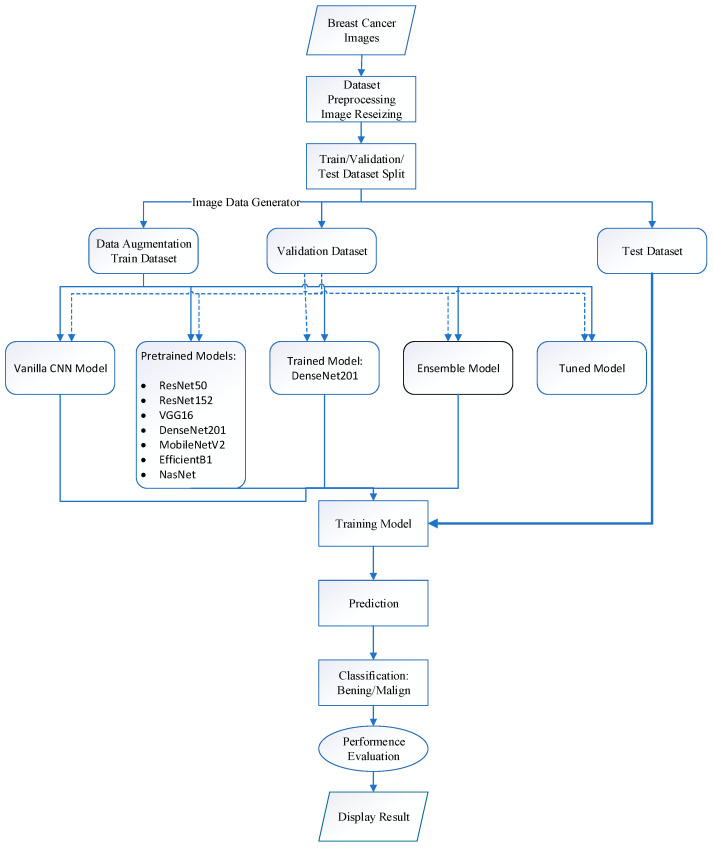
Breast tumor dataset model flow chart.

Images were generated with Image Data Generator and fed into the “flow_from_directory” function in batches of 32, with “categorical” as the classification method for training, validation, and testing (Figure 3).

### 3.2. Convolutional Neural Network (CNN) Model

**Figure 4:**
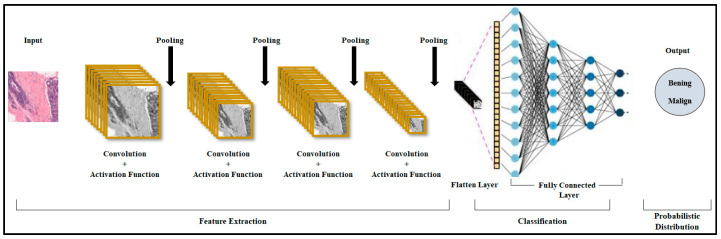
Single-layer convolutional neural network.

CNNs are a specialized type of artificial neural network designed primarily for processing structured data, such as images and videos [8]. CNNs are highly effective for tasks such as image classification, object detection, and segmentation due to their ability to automatically and adaptively learn spatial hierarchies of features from data. The architecture of the CNN model is as shown in Figure 4.

Convolutional layers apply convolution operations to the input data. A convolution operation involves a kernel (filter) sliding across the input, extracting spatial features such as edges, textures, or patterns. It outputs a feature map, representing the presence of learned features at different spatial positions. Activation functions are applied after convolution to introduce non-linearity. Pooling layers reduce the spatial dimensions of feature maps, retaining essential information and reducing the computational complexity. Fully connected layers flatten the feature maps into one-dimensional vectors and pass them through dense layers for the final decision-making. Typically used at the end of the network for classification or regression tasks, the output layer converts the outputs of the network into probabilities for classification tasks [43].

### 3.3. ResNet50 Architecture

ResNet50 is a deep convolutional neural network (CNN) that forms part of the ResNet (Residual Network) family. It is distinguished by its introduction of residual learning, which addresses the vanishing gradient problem in training very deep networks. The network comprises 50 layers, including convolutional, pooling, and fully connected layers, along with shortcut (residual) connections [44]. These residual connections enable the network to learn identity mappings, allowing deeper architectures to be constructed without a corresponding reduction in performance. ResNet50 is a prevalent tool in the field of computer vision, employed in tasks such as image classification, object detection, and segmentation. Its popularity can be attributed to two key factors: an efficient architectural design and a high level of accuracy. The network comprises multiple residual blocks, each comprising convolutional layers and batch normalization, followed by a rectified linear unit (ReLU) activation function. The network’s modularity and transfer learning capabilities render it suitable for applications in medical imaging. Pre-trained versions of ResNet50 are commonly used as a starting point for various tasks, leveraging their robust feature extraction capabilities [45].

### 3.4. ResNet152 Architecture

ResNet152 is a deep convolutional neural network (CNN) from the Residual Network (ResNet) family, featuring 152 layers. It builds on the success of ResNet50 by significantly increasing depth, thereby enhancing the network’s ability to capture complex features while mitigating issues such as the vanishing gradient problem through the use of residual connections [46]. Although ResNet152 offers higher accuracy than shallower versions like ResNet50, it comes at the cost of increased computational complexity and memory requirements. However, its pre-trained models make it a popular choice for transfer learning, particularly in applications like medical imaging, satellite image analysis, and advanced facial recognition [47].

### 3.5. VGG16 Architecture

VGG16 is a popular convolutional neural network (CNN) architecture introduced by the Visual Geometry Group (VGG) at the University of Oxford. VGG16 comprises 16 layers, including 13 convolutional layers and 3 fully connected layers, with interspersed pooling layers [48]. It is widely acknowledged for its simplicity and efficacy in computer vision tasks, including image classification, object detection, and feature extraction.

The network uses small (3 × 3) convolutional filters throughout, which helps capture intricate details in images while reducing the computational complexity. It employs a consistent depth of layers, with the convolutional layers followed by max-pooling layers to progressively reduce the spatial dimensions.

### 3.6. DenseNet201 Architecture

DenseNet201 is a deep convolutional neural network (CNN) architecture from the DenseNet (Densely Connected Networks) family. It was designed by [49] with the objective of enhancing feature reuse and gradient flow in deep networks. It contains 201 layers, including convolutional, pooling, and fully connected layers, interconnected through dense blocks. DenseNet201′s efficient use of parameters and strong gradient propagation make it an attractive choice for tasks requiring deep feature extraction while managing computational costs.

### 3.7. MobileNetV2 Architecture

MobileNetV2 is a convolutional neural network (CNN) architecture that has been optimized for use on mobile and embedded devices, offering a lightweight and efficient solution. Sandler et al. introduced this architecture in 2018, building upon the original MobileNet to achieve a balance between accuracy and computational efficiency. This makes it suitable for real-time applications in resource-constrained environments. MobileNetV2 is parameterized by two factors: a width multiplier (which reduces the number of channels) and a resolution multiplier (which scales the input resolution), allowing customization based on device constraints.

### 3.8. EfficientNet-B1 Architecture

EfficientNet-B1 is one of the models from the EfficientNet family, which is a series of convolutional neural networks (CNNs) designed to achieve high accuracy while maintaining computational efficiency. Introduced by [50], EfficientNet-B1 is part of a scaling framework that uniformly balances the three dimensions of a neural network: depth, width, and resolution. This balance is achieved using a compound scaling method, which scales these dimensions proportionally to improve performance. The Swish activation function is employed, which provides a more gradual gradient flow than traditional functions such as ReLU, thereby facilitating superior optimization.

NASNet (Neural Architecture Search Network) is a deep learning architecture discovered through the use of Neural Architecture Search (NAS), a technique that automates the design of neural networks. In 2018, ref. [51] introduced NASNet, which leverages reinforcement learning to identify optimal architectural building blocks that maximize accuracy and efficiency. NAS is employed to automatically explore and design network architectures, thereby reducing the necessity for manual tuning. The system utilizes a search space that encompasses convolutional layers, pooling layers, and other operations.

### 3.9. Ensemble Model

An ensemble model is a machine learning approach that combines the predictions of multiple models with the objective of improving the overall performance and robustness [52]. The underlying concept is to leverage the strengths of individual models while mitigating their weaknesses. Ensemble methods are particularly effective in increasing accuracy, reducing overfitting, and making predictions more robust to noise.

Ensemble methods work best when the base models are diverse, meaning that they make different kinds of errors. It combines the outputs of base learners. The method of aggregation (e.g., majority voting, averaging, weighted sum) depends on the ensemble approach.

### 3.10. Parameter Optimization

In deep learning, hyperparameters are unique parameters that guide the model’s learning process and require careful adjustment. These parameters determine the structure of the neural network and the training dynamics, significantly impacting the model’s success. Properly optimizing hyperparameters can enhance the model’s overall performance. In recent years, optimizing parameters has become crucial in developing deep learning models due to the growing number of neural networks aimed at achieving optimal accuracy with fewer weights and parameters. Since selecting appropriate hyperparameters is challenging, aligning them with experimental values is equally complex. Hyperparameter tuning is a sophisticated process requiring careful design. For commonly used models, researchers often set hyperparameters manually, leveraging insights from prior studies. While manual adjustment works well for smaller-scale models, larger or newly introduced models demand extensive experimentation to determine the most effective hyperparameters [53].

Hyperparameters can be categorized into two main groups: those that influence model training and those that define the model’s design. The selection of the right training hyperparameters helps neural networks learn more efficiently and achieve a better performance. Commonly used optimization algorithms for training deep neural networks include momentum, stochastic gradient descent, AdaGrad, RMSprop, and Adam. Among these, the learning rate and batch size are particularly critical, as they directly impact the neural network’s convergence speed during training. On the other hand, hyperparameters for model design are more concerned with the architecture of the neural network. Examples include the number of hidden layers and the width of those layers. These values play a key role in determining the network’s overall performance. To elaborate, these parameters must be carefully chosen to ensure optimal results [54].

Learning Rate: This hyperparameter determines how much the network’s weights are adjusted during training. While a high learning rate can lead to quicker weight updates, it risks overshooting the optimal values. Conversely, a low learning rate slows the learning process but ensures more precise updates. Typically, the learning rate needs manual tuning throughout the training process, as this adjustment is critical in achieving high accuracy.

Epoch Count: An epoch refers to one complete pass of the entire training dataset through the model. The number of epochs dictates how often the model processes all training data. Too many epochs can result in overfitting, where the model performs well on training data but poorly on unseen data, while too few epochs may prevent the model from fully learning the patterns in the data.

Mini-Batch Size: The batch size is the number of samples used in each training iteration. Smaller batch sizes can speed up training but may impact the overall model performance. Mini-Batches, often utilized in the Probabilistic Gradient Projection Algorithm, are randomly generated subsets of training data, with gradient calculations performed on these subsets.

Hyperparameter tuning is typically a trial-and-error process, involving testing various combinations of hyperparameter values to find the one that delivers the best performance. This iterative process is essential in improving the model’s generalizability and minimizing the risk of overfitting to the training dataset [4].

### 3.11. Evaluation Criteria

Artificial intelligence applications operate based on the principles of trial, feedback, adjustment, and outcome. Prior to conducting research, a model is developed, and its validity is evaluated through feedback. Subsequently, necessary improvements are implemented, and the model is refined to achieve the desired level of accuracy. Test outcomes are assessed using various metric values, and the model’s performance is determined based on these results. Evaluation criteria play a crucial role in comparing different models and differentiating their results.

To estimate success rates in classification processes, various performance metrics are utilized. Among these, the most widely used criterion in classification problems is the accuracy (ACC) metric. However, accuracy alone does not always provide definitive insights. For a more precise and reliable analysis, additional metrics must be employed. A review of the literature reveals that, alongside accuracy, other metrics such as precision (Prec), sensitivity (recall), and the F1 score (F1) are frequently used. These values can be calculated in a matrix format by utilizing the confusion matrix. The confusion matrix allows the computation of true positive (TP), true negative (TN), false positive (FP), and false negative (FN) values from classification results [55]. Table 1 illustrates the components of the confusion matrix.

$$\;\;\;\mathrm{Accuracy}=\frac{TP+TN}{TP+FP+FN+TN}\;\;\;\;\mathrm{Recall}=\frac{TP}{TP+FN}$$

$$\mathrm{Precision}=\frac{TP}{TP+FP}\;\;\;\;\;\;\;F1\;\mathrm{score}=\frac{2xPrecision*Recall}{Precision+Recall}$$

A confusion matrix is a table that is frequently used to calculate how well a classification system performs numerically on a test dataset with known real values.

### 3.12. Experimental Results and Discussion

In this part of the study, first of all, it is explained how the models used were created. Many different models were run using a trial-and-error method, and the models that gave the highest accuracy rates were selected. In addition, the parameter optimization applied in these models and changes in the model architectures in accordance with the data were also tested by the trial-and-error method. In addition, the classification results for benign and malignant tumors for the dataset described in the Materials and Methods Section are shown.

Starting with the CNN model, the Vanilla model had an input layer with a shape of (50, 50, 3). The first layer of hidden layers was the Conv2D layer with a filter count of 32 and a kernel size of 3 × 3. The Maxpool2D neuron has a pool size of 4 × 4, and a dropout layer with three arguments followed. Next, another Conv2D layer with a filter count of 32 and a kernel size of 3 × 3 was used. The Maxpool2D neuron has a pool size of 3 × 3 and a dropout layer. The neuron has three arguments and a dropout layer. Three arguments followed. A flatten layer followed by a dense layer with 64 neurons with “Relu” as the activation function was used as the last layer of the hidden layers. The output layer consisted of a dense layer with 2 neurons with the activation function “SoftMax”. For optimization, the Adam method was used, and categorical cross-entropy was used for the loss function. The whole network had nine layers with 28,770 trainable parameters. Early stopping was used to prevent overfitting, and the patience parameter had an argument of five; when the model did not improve after five epochs, training would stop.

We included pre-trained models from Keras’s application module as subsequent models. ResNet50, ResNet152, VGG16, DenseNet201, MobileNetV2, EfficientB1, and NasNet were the pre-trained models used. We used weights derived from the “ImageNet” library. We used a pre-trained model as the output layer. This was followed by a flatten layer and a dense layer with 32 neurons and a “Relu” activation function. The dropout layer with three arguments was the last layer in the hidden layers, and the output layer with two neurons and “SoftMax” activation was used. The pre-trained model with the highest accuracy was selected and retrained with random variables with the same architecture if retraining with our dataset, including pathological specimens, which showed improvement compared to the pre-trained model trained with images from “ImageNet”.

We included the five models with the highest accuracy rates in an ensemble model and selected the average for aggregation. Finally, we optimized the hyperparameters of the Vanilla model. The first Conv2D layer had 32, 48, or 64 filters and a kernel size of 3 × 3, 4 × 4, or 5 × 5. It also had a dropout layer with different parameters: 0.2, 0.3, 0.4, or 0.5. The second Conv2D layer had 64, 128, 192, or 256 filters and a kernel size of 3 × 3, 4 × 4, or 5 × 5. It also had a dropout layer with different parameters: 0.2, 0.3, 0.4, or 0.5. It had a dense layer with different neurons and activation functions: 64, 128, 192, or 256, “Relu” or “Tanh.” We used either Adam or stochastic gradient descent algorithms for the loss function. We considered a total of 18,432 possible combinations and randomly selected 10 for tuning. The model with the highest accuracy was used for the final model. We evaluated the models’ fitness using separate training and validation accuracy loss plots. Each model’s accuracy, precision, recall, F1 score, ROC curve with AUC score, and precision–recall curve with AUC-PR underwent performance testing. We used Python version 3.12.3 with TensorFlow version 2.18.0, scikit-learn 1.5.2, and panda’s version 2.2.3 for data processing, model building, and testing.

**Figure 5:**
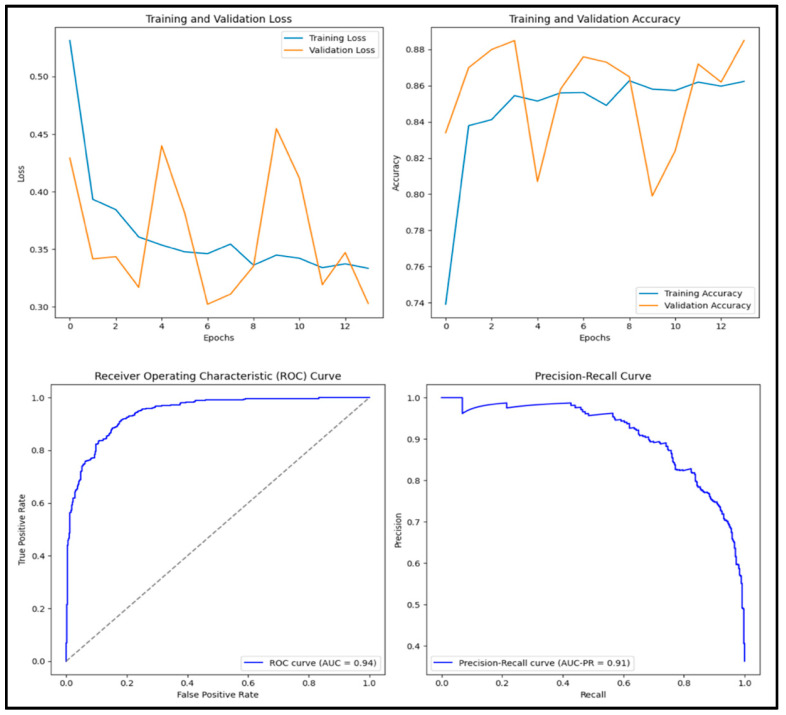
Training and validation loss and accuracy graphs with ROC curve and precision–recall curves for Vanilla CNN model.

The model classified 3828 (38.3%) of the 10,000 images as benign and 6172 (61.7%) as malignant. The Vanilla CNN model had an accuracy score of 0.854, a precision score of 0.788, a recall score of 0.857, an F1 score of 0.821, and an AUC score of 0.937. Of the 1000 images tested, 335 (33.5%) were true positives, 519 (51.9%) were true negatives, 90 (0.9%) were false positives, and 6 (0.6%) were false negatives. Figure 5 displays training and validation loss and accuracy graphs along with the ROC curve and precision–recall curves.

The pre-trained ResNET-50 model had an accuracy score of 0.609, a precision score of 0, a recall score of 0, an F1 score of 0, and an AUC score of 0.602. Of the 1000 images tested, 0 (0%) were true positive, 609 (60.9%) were true negative, 0 (0%) were false positive, and 391 (39.1%) were false negative.

**Figure 6:**
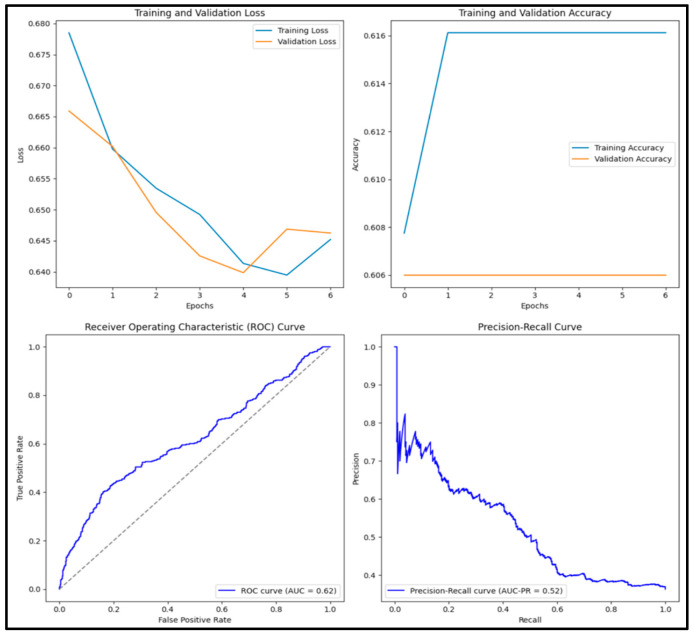
Training and validation loss and accuracy graphs with ROC curve and precision–recall curves for pre-trained ResNET-50 model.

The pre-trained ResNET-152 model shown in Figure 6 has an accuracy score of 0.609, precision score of 0, recall score of 0, F1 score of 0 and AUC score of 0.752. Out of 1000 images tested, 0 (0%) were true positive, 609 (60.9%) were true negative, 0 (0%) were false positive and 391 (39.1%) were false negative.

**Figure 7:**
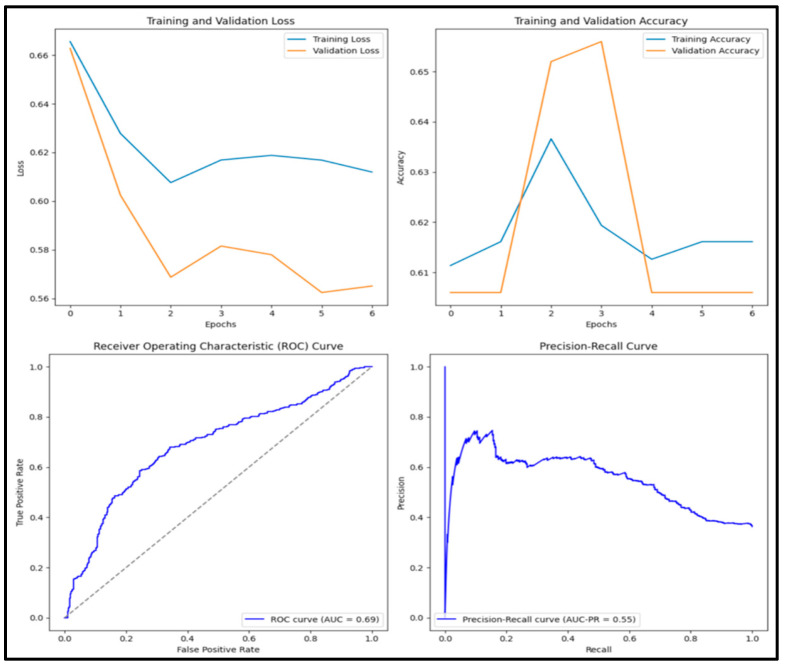
Training and validation loss and accuracy graphs with ROC curve and precision–recall curves for pre-trained ResNET-152 model.

In Figure 7, the accuracy score of the pre-trained VGG16 model is 0.794, precision score is 0.790, recall score is 0.645, F1 score is 0.710 and AUC score is 0.853. Of the 1000 images tested, 252 (25.2%) were true positive, 542 (54.2%) were true negative, 67 (0.7%) were false positive and 139 (13.9%) were false negative.

**Figure 8:**
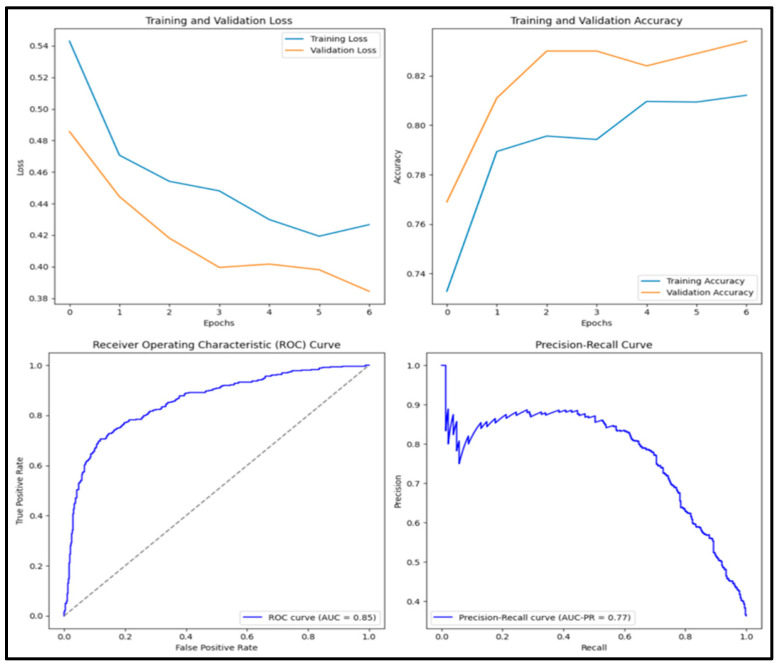
Training and validation loss and accuracy graphs with ROC curve and precision–recall curves for pre-trained VGG16 model.

In Figure 8, the pre-trained DenseNet152 model has an accuracy score of 0.818, precision score of 0.785, recall score of 0.737, F1 score of 0.760 and AUC score of 0.882. Of the 1000 images tested, 288 (28.8%) were true positive, 530 (53.0%) were true negative, 79 (0.8%) were false positive and 103 (10.3%) were false negative.

**Figure 9:**
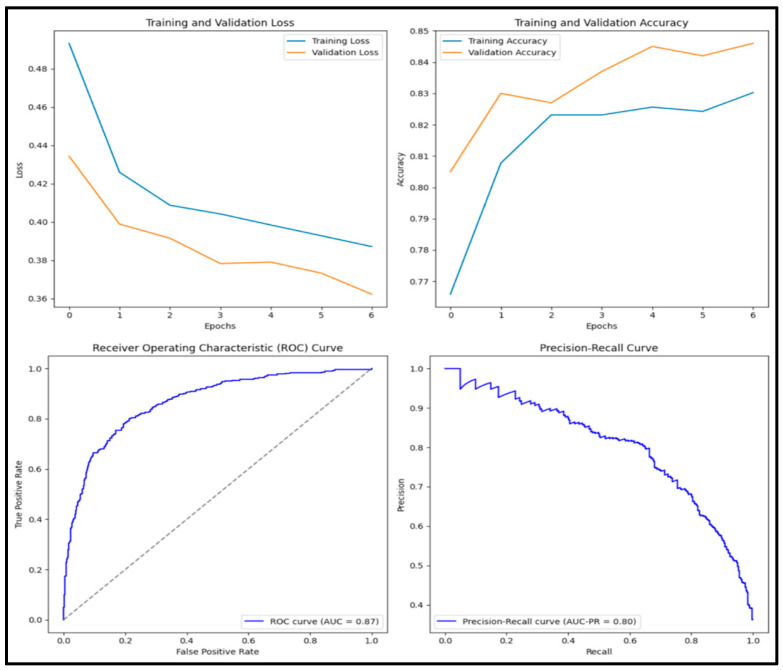
Training and validation loss and accuracy graphs with ROC curve and precision–recall curves for pre-trained DenseNet152 model.

In Figure 9, the accuracy score of the pre-trained MobileNetV2 model is 0.773, precision score is 0.693, recall score is 0.755, F1 score is 0.722 and AUC score is 0.856. Of the 1000 images tested, 295 (29.5%) were true positive, 478 (47.8%) were true negative, 131 (13.1%) were false positive and 96 (1.0%) were false negative.

**Figure 10:**
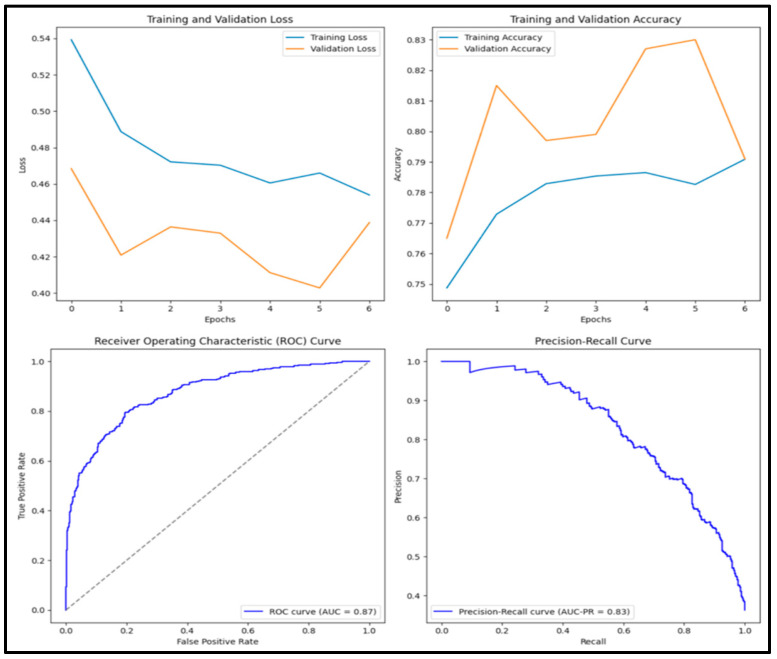
Training and validation loss and accuracy graphs with ROC curve and precision–recall curves for pre-trained MobileNetV2 model.

In Figure 10, the pre-trained EfficientB1 model has an accuracy score of 0.609, precision score of 0, recall score of 0, F1 score of 0 and AUC score of 0.472. Out of 1000 images tested, 0 (0%) were true positive, 609 (60.9%) were true negative, 0 (0%) were false positive and 391 (39.1%) were false negative.

**Figure 11:**
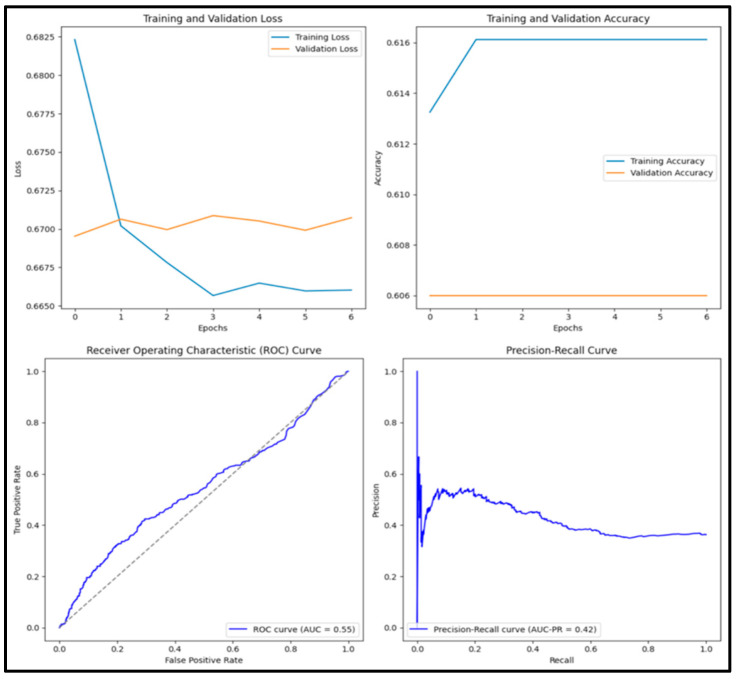
Training and validation loss and accuracy graphs with ROC curve and precision–recall curves for pre-trained EfficientB1 model.

In Figure 11, the accuracy score of the pre-trained NasNet Large model is 0.705, precision score is 0.648, recall score is 0.537, F1 score is 0.587 and AUC score is 0.762. Of the 1000 images tested, 210 (21%) were true positive, 495 (49.5%) were true negative, 114 (11.4%) were false positive and 181 (18.1%) were false negative.

**Figure 12:**
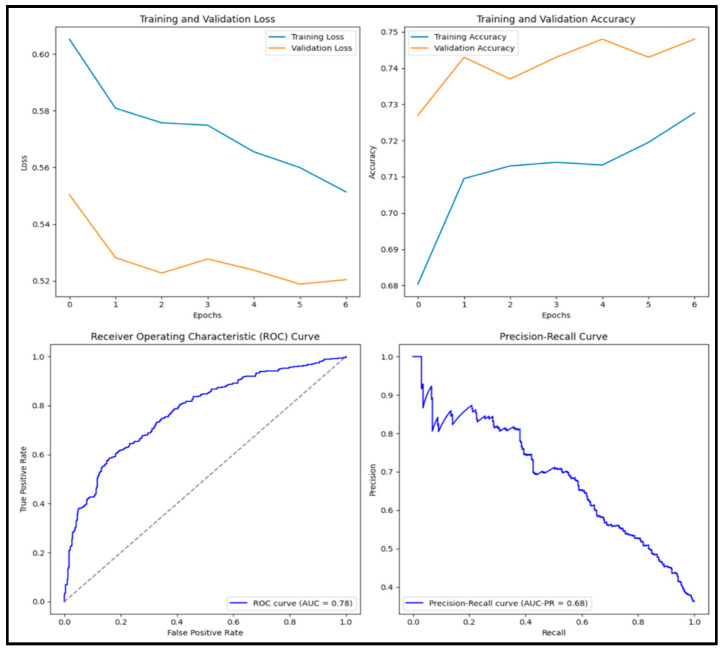
Training and validation loss and accuracy graphs with ROC curve and precision–recall curves for pre-trained NasNet Large model.

The DenseNet201 model we trained in Figure 12 has an accuracy score of 0.894, precision score of 0.882, recall score of 0.841, F1 score of 0.861 and AUC score of 0.958. Of the 1000 images tested, 329 (32.9%) were true positive, 565 (56.5%) were true negative, 44 (0.4%) were false positive and 62 (0.6%) were false negative.

**Figure 13:**
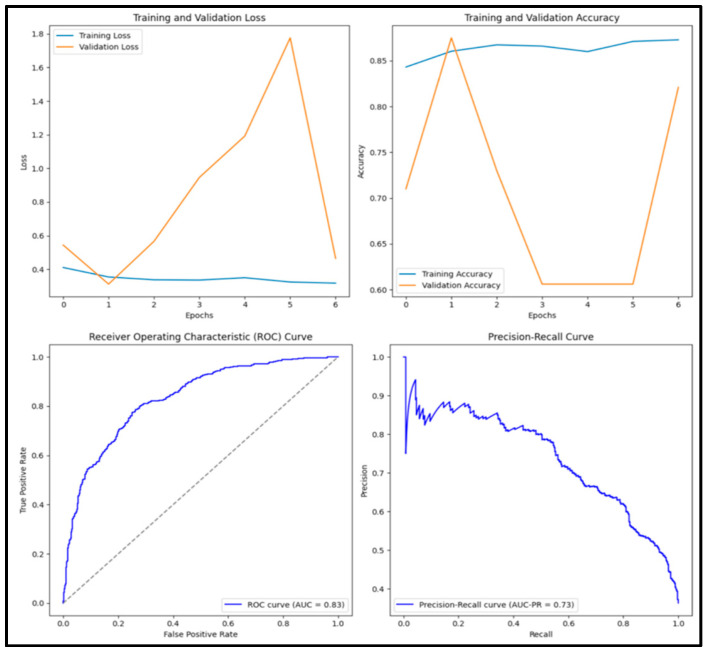
Training and validation loss and accuracy graphs with ROC curve and precision–recall curves for trained DenseNet201 model.

In Figure 13, the ensemble model including Vanilla model, pre-trained VGG16, DenseNet152, mobilenetV2 and NasNET has an accuracy score of 0.822, precision score of 0.728, recall score of 0.870, F1 score of 0.793 and AUC score of 0.917. Of the 1000 images tested, 340 (34.0%) were true positive, 482 (48.2%) were true negative, 127 (12.7%) were false positive and 51 (0.5%) were false negative.

**Figure 14:**
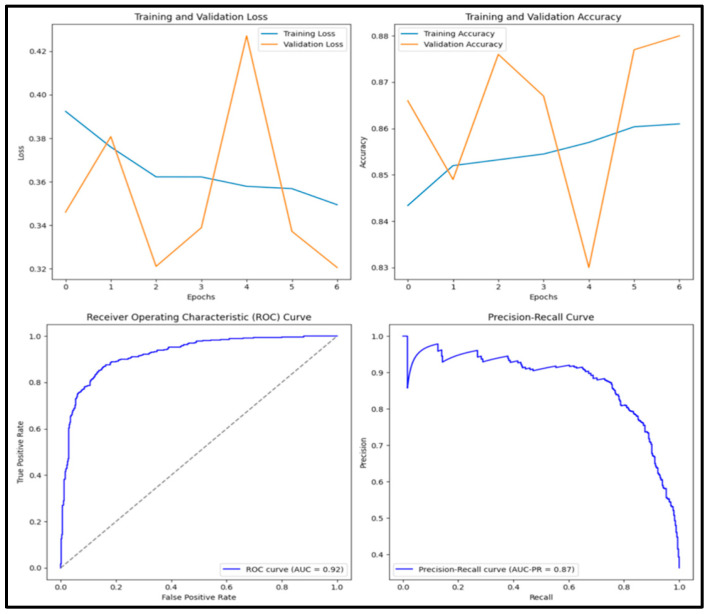
Training and validation loss and accuracy graphs with ROC curve and precision–recall curves for ensemble model.

In Figure 14, the optimized model with hyperparameter tuning has an accuracy score of 0.820, precision score of 0.762, recall score of 0.785, F1 score of 0.773 and AUC score of 0.885. Of the 1000 images tested, 307 (30.7%) were true positive, 513 (51.3%) were true negative, 96 (1.0%) were false positive and 84 (0.8%) were false negative.

**Figure 15:**
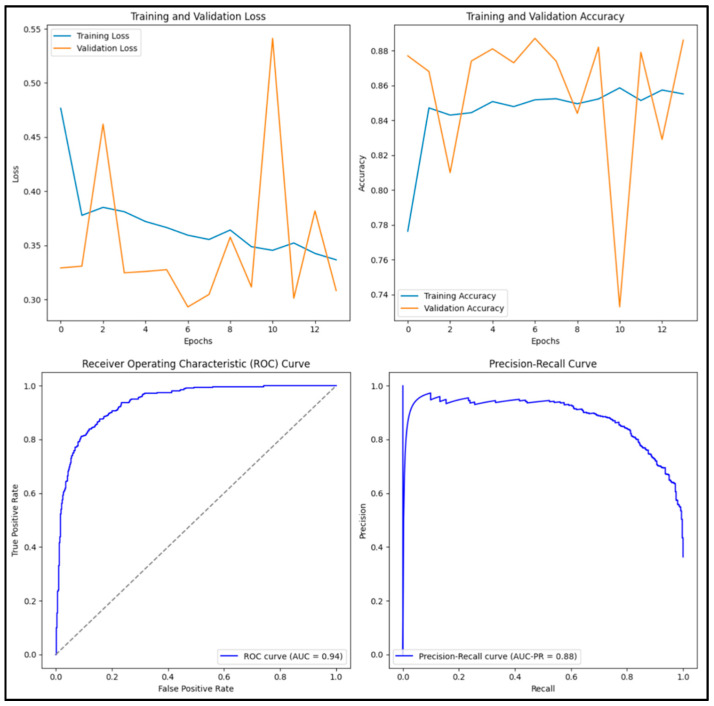
Training and validation loss and accuracy graphs with ROC curve and precision–recall curves for optimized model.

Table 2 shows the metrics of the models, such as the accuracy, precision, sensitivity, F1 score, and AUC. In Figure 15, the Vanilla CNN model is a standard CNN model that captures features in the image using basic convolutional layers. Although it is a simple model built with simple and basic features, it performed effectively in classification. In our analyses and comparisons, the DenseNet201 model provided deeper but more optimized learning than the other models and stood out as the most successful model in breast cancer classification. The most important reason for this is that it is a deep neural network architecture that increases learning efficiency by establishing direct connections between layers. One of the main factors in the success of this model is that it optimizes the flow of information and improves the learning process thanks to its dense connection structure. Unlike traditional CNN structures, each layer receives input from all previous layers, increasing the parameter efficiency and reducing the vanishing gradient problem. It also increases the reuse of features in the deep layers, allowing for more effective representation learning.

The ensemble learning model created by combining multiple models (Vanilla, VGG16, DenseNET152, MobilenetV2, and NasNet) increases the overall success rate by combining the advantages of different architectures. Another remarkable finding of the study is that the ensemble model could not match the performance of the individual best model. The main reason for this is that the ensemble model is more generalizable, but the individual best model can perform better in individual tasks. In addition, low-performing individual models within the ensemble model may have lowered the average result. This finding suggests that only high-performing models should be combined in order for ensemble methods to be successful. Finally, the model developed with hyperparameter optimization, although not as successful as DenseNet201, has shown results that have the potential to improve the generalization performance. However, it is clear that the risk of overfitting should be reduced during hyperparameter selection, and more experiments are needed to improve the overall performance. In addition, a model developed here with hyperparameter optimization was CNN-based and improved by optimizing certain parameters. However, the success level was lower than expected due to the fact that optimization increases the risk of overfitting and certain hyperparameter combinations reduce the generalizability of the model. Choosing more generalizable values in the selection of hyperparameters may lead to better performance in the optimized model. Furthermore, the main reasons for the low accuracy and performance metrics of the other models include incompatibility with the dataset, insufficient transfer learning, and architectural limitations. The ResNet50, ResNet152, and EfficientB1 models were not adapted well enough in the transfer learning phase. These models were not customized for medical images such as breast cancer images and were not sensitive enough to specific medical data, as they were optimized for general image recognition.

Despite promising results, several challenges remain in deploying deep learning models in real-world clinical settings. One critical issue is the interpretability of deep learning predictions, as black-box models may lack transparency in decision-making, leading to concerns in clinical practice. Furthermore, generalizability remains a key challenge, as models trained on specific datasets may struggle with variations in imaging protocols across different institutions. To improve clinical applicability, future research should focus on explainable AI techniques, domain adaptation strategies, and collaborative efforts between AI researchers and healthcare professionals. By refining these models and integrating them into radiological workflows, deep learning can play a crucial role in enhancing early breast cancer detection and patient outcomes.

## 4. Conclusions

Early diagnosis remains one of the most critical components in the effective management and treatment of cancer patients. This study addresses this clinical imperative by proposing a deep learning-based approach to the classification of breast cancer using histopathological images. We used models that effectively differentiated between benign and malignant cases, leveraging advanced neural network architectures to extract meaningful features from medical images. The histopathological images used in the study show that high accuracy, sensitivity, and specificity can be achieved when images are classified using deep learning models. However, while the model exhibits strong classification capabilities, its reliability is influenced by factors such as dataset quality, class imbalance, and image resolution. Addressing these limitations through data augmentation, transfer learning, and more diverse training datasets can further enhance the robustness of the system. In conclusion, the study offers a thorough and novel method of classifying and diagnosing breast tumors through artificial intelligence, specifically using convolutional neural networks (CNNs) like ensemble, Tuned Model, VGG16, MobileNetv2, DenseNet, Vanilla, ResNet, and others.

The results demonstrate that DenseNet201 achieved the highest classification accuracy of 89.4%, with a precision of 88.2%, recall of 84.1%, F1 score of 86.1%, and AUC score of 95.8%. The superior performance of DenseNet201 can be attributed to its dense connectivity, which optimizes feature reuse, mitigates the vanishing gradient problem, and enhances deep feature extraction. While the ensemble model combining multiple architectures provided competitive results, it did not surpass the performance of the best individual model, suggesting that the careful selection of high-performing architectures is essential in order for ensemble learning to be effective.

Hyperparameter tuning significantly contributed to model optimization, demonstrating that a well-calibrated balance between complexity and generalization is key in achieving superior performance. However, excessive fine-tuning led to overfitting risks, reinforcing the need for systematic optimization approaches. Moreover, transfer learning models such as ResNet and EfficientNet underperformed, likely due to their lack of specific adaptation for medical imaging tasks. This underscores the importance of fine-tuning pre-trained models on domain-specific datasets to enhance their diagnostic capabilities.

In conclusion, this study underscores the potential of deep learning in breast cancer classification and highlights the importance of selecting the right model architectures, applying effective hyperparameter tuning, and leveraging ensemble strategies judiciously. Future research should focus on integrating multi-modal imaging data, refining ensemble learning methodologies, and expanding dataset diversity to further improve classification accuracy and real-world clinical applicability. Additionally, exploring interpretability techniques for deep learning models could enhance their adoption in clinical practice by providing transparent and explainable decision-making processes.

## Figures and Tables

**Table 1:** Confusion matrix.

		Predicted Value	
		Positive	Negative
**Actual Value**	Positive	True positive (TP)	False negative (FN)
	Negative	False positive (FP)	True negative (TN)

**Table 2:** Accuracy, precision score, recall score, F1 score, and AUC of model.

Model	Accuracy	Precision	Recall	F1	AUC
Vanilla	0.854	0.788	0.857	0.821	0.937
ResNet50	0.609	0	0	0	0.602
ResNet152	0.609	0	0	0	0.752
VGG16	0.794	0.790	0.645	0.710	0.853
DenseNet152	0.818	0.785	0.737	0.760	0.882
MobileNetv2	0.773	0.693	0.755	0.722	0.856
EfficientB1	0.609	0	0	0	0.472
NasNet	0.705	0.648	0.537	0.587	0.762
DenseNet201	0.894	0.882	0.841	0.861	0.958
Ensemble	0.822	0.728	0.870	0.793	0.917
Tuned Model	0.820	0.762	0.785	0.773	0.885

## Data Availability

Data are available from 2025 [42] (https://www.kaggle.com/code/paultimothymooney/predict-idc-in-breast-cancer-histology-images/notebook (accessed on 7 July 2025)).
